# Evaluation of Blood-Based Antibody Rapid Testing for HIV Early Therapy: A Meta-Analysis of the Evidence

**DOI:** 10.3389/fimmu.2018.01458

**Published:** 2018-06-26

**Authors:** Xiaojie Huang, Xinchao Liu, Jieqing Chen, Yugang Bao, Jianhua Hou, Xiaofan Lu, Wei Xia, Huan Xia, Aixin Song, Zhiying Liu, Bin Su, Hui Chen, Yaokai Chen, Hao Wu

**Affiliations:** ^1^Center for Infectious Diseases, Beijing You’an Hospital, Capital Medical University, Beijing, China; ^2^Department of Infectious Diseases, Peking Union Medical College Hospital, Beijing, China; ^3^Department of Information Technology, Peking Union Medical College Hospital, Beijing, China; ^4^AIDS Healthcare Foundation, Beijing, China; ^5^School of Biomedical Engineering, Capital Medical University, Beijing, China; ^6^Department of Infectious Diseases, Chongqing Public Health Medical Center, Chongqing, China; ^7^Zunyi Medical University, Zunyi, China

**Keywords:** western blot, rapid test, HIV, early therapy, meta-analysis

## Abstract

**Background:**

Western blot (WB) assay is considered the gold standard test for HIV infection confirmation. However, it requires technical expertise and is quite time-consuming. WHO recommends blood-based rapid diagnosis to achieve same-day test and treatment. However, this rapid testing strategy has not been promoted worldwide due to inadequate research evaluating the effectiveness of rapid tests (RTs) as an alternative confirmatory HIV test for WB. This study aims to compare the diagnostic performance of rapid HIV tests compared with WB.

**Methods:**

PubMed and Web of Science were searched for publications on rapid HIV tests using blood specimen. A meta-analysis was performed to quantitatively evaluate the diagnostic performance of rapid HIV tests compared with the WB assay in terms of pooled sensitivity, specificity, area under summary receiver operating characteristic (SROC) curve, and diagnostic odds ratio (DOR).

**Results:**

Twenty articles involving 27,343 fresh specimens for rapid HIV tests were included in the meta-analysis. Regarding Capillus HIV-1/HIV-2, the pooled sensitivity, specificity, area under SROC curve, and DOR derived from six studies were 0.999 (95% CI, 0.956–1.000), 0.999 (95% CI, 0.991–1.00), 1.00 (95% CI, 0.99–1.00), and 1.0 × 10^6^ (95% CI, 2.6 × 10^4^–3.9 × 10^7^) compared with the WB assay, respectively. With respect to Determine HIV-1/2, the pooled sensitivity, specificity area under SROC, and DOR derived from eight studies were 1.00 (95% CI, 0.789–1.000), 0.992 (95% CI, 0.985–0.996), 1.00 (95% CI, 0.99–1.00), and 1.8 × 10^6^ (95% CI 406.049–7.8 × 10^9^) compared with the WB assay, respectively. Regarding two-step serial RTs, the pooled sensitivity, specificity area under SROC, and DOR derived from eight studies were 0.998 (95% CI, 0.991–1.000), 0.998 (95% CI, 0.994–0.999), and 1.00 (95% CI 0.99–1.00) compared with the WB assay, respectively.

**Conclusion:**

Our meta-analysis results may provide evidenced-based support for substituting RT for WB. Blood-based rapid HIV tests have comparable sensitivity and specificity to WB for HIV early therapy.

## Introduction

Western blot (WB) is regarded as the gold standard for a definitive confirmatory test for HIV infection. It usually takes up to 7–14 days to get the result and costs a lot (1, 2). Moreover, WB tests can only be performed by lab-professionals, which greatly restrict the accessibility of HIV testing. Surveys indicate that roughly 40–50% of HIV cases progressed to AIDS within the first year of diagnosis largely due to late diagnosis (3, 4). According to the Chinese integrated HIV/AIDS database, about 25% of newly diagnosed HIV/AIDS cases reported in China were already AIDS patients when they were first identified (5). Such late diagnoses are mainly caused by lack of access to fast and accurate HIV testing, particularly in settings that continue to rely on traditional WB confirmatory testing (6, 7). However, simple and rapid blood-based HIV tests other than ELISA that target HIV antibodies have become available, enabling testing in outreach settings outside the laboratory at the point of client contact (8). Compared with WB assay, a rapid and accurate diagnosis in 15–30 min strategy is required. Blood-based rapid tests (RTs) targeting HIV antibody are convenient tests that require minimal laboratory infrastructure and expertise training compared with WB assay. Test results can be available in less than 30 min, so WHO recommends its serial use in different clinical settings to achieve same-day test and treatment (9, 10). These tests can offer prompt test results and receipt of a “preliminary positive” test result, and therefore have greatly enhanced both the awareness rate and result notification rate of the infected and the coverage of antiretroviral therapy (ART) (11). The FDA recommends that the sensitivity and specificity of RTs should exceed 98% (12). Health authorities have recommended diagnosis strategies to replace traditional WB confirmatory test (13–15), using either combinations of HIV antibody RT reagents or combinations of RT reagents and ELISA reagents.

China relies on a traditional HIV testing algorithm that requires WB confirmation to date. It is of great need to explore a fast and accurate strategy of HIV diagnosis by using RT centered testing. However, the effectiveness of HIV diagnosis using RTs has not been adequately explored. Therefore, the purpose of this paper is to evaluate the diagnostic performance of blood-based rapid HIV tests compared with the WB assay in terms of pooled sensitivity and specificity by meta-analysis, aiming to summarize the overall diagnostic accuracy of blood-based RTs as alternative algorithms for HIV early therapy.

## Methods

This study aims to compare the diagnostic performance of rapid HIV tests compared with WB assay in terms of pooled sensitivity, specificity, area under summary receiver operating characteristic (SROC) curve, and diagnostic odds ratio (DOR) among different populations. The work was reported in accordance with the Preferred Reporting Items for Systematic Reviews and Meta-Analysis PRISMA guidelines [see RPISMA checklist in Supplementary Material (16)].

### Search Strategy

PubMed and Web of Science were searched using combinations of the following search keywords: [(HIV or AIDS) and (“rapid test” or “fast test”)]. Reference lists of selected articles and related review articles were further screened. Additional searches in Google Scholar and manual searches of related journals (e.g., AIDS; Journal of Clinical Virology and Lancet Infectious Diseases) were also performed. Articles were limited to those published from January 2000 to May 2016. The search was limited to English language publications.

### Study Selection

Studies were identified as eligible if RTs were conducted on whole blood sample.

Studies were excluded if (i) sensitivity, specificity, positive predictive value (PPV), and negative predictive value (NPV) were not reported or could not be calculated from the information provided; (ii) RTs were conducted on saliva; (iii) RT results were not confirmed by WB; or (iv) they were the reviews of rapid HIV antibody tests.

Two independent reviewers (Jieqing Chen and Xiaojie Huang) without prior consideration of the results initially selected search results based on titles and abstracts. The remaining articles were further selected by full-text assessment. Disagreements between reviewers about eligibility were resolved by discussion.

**Figure 1:**
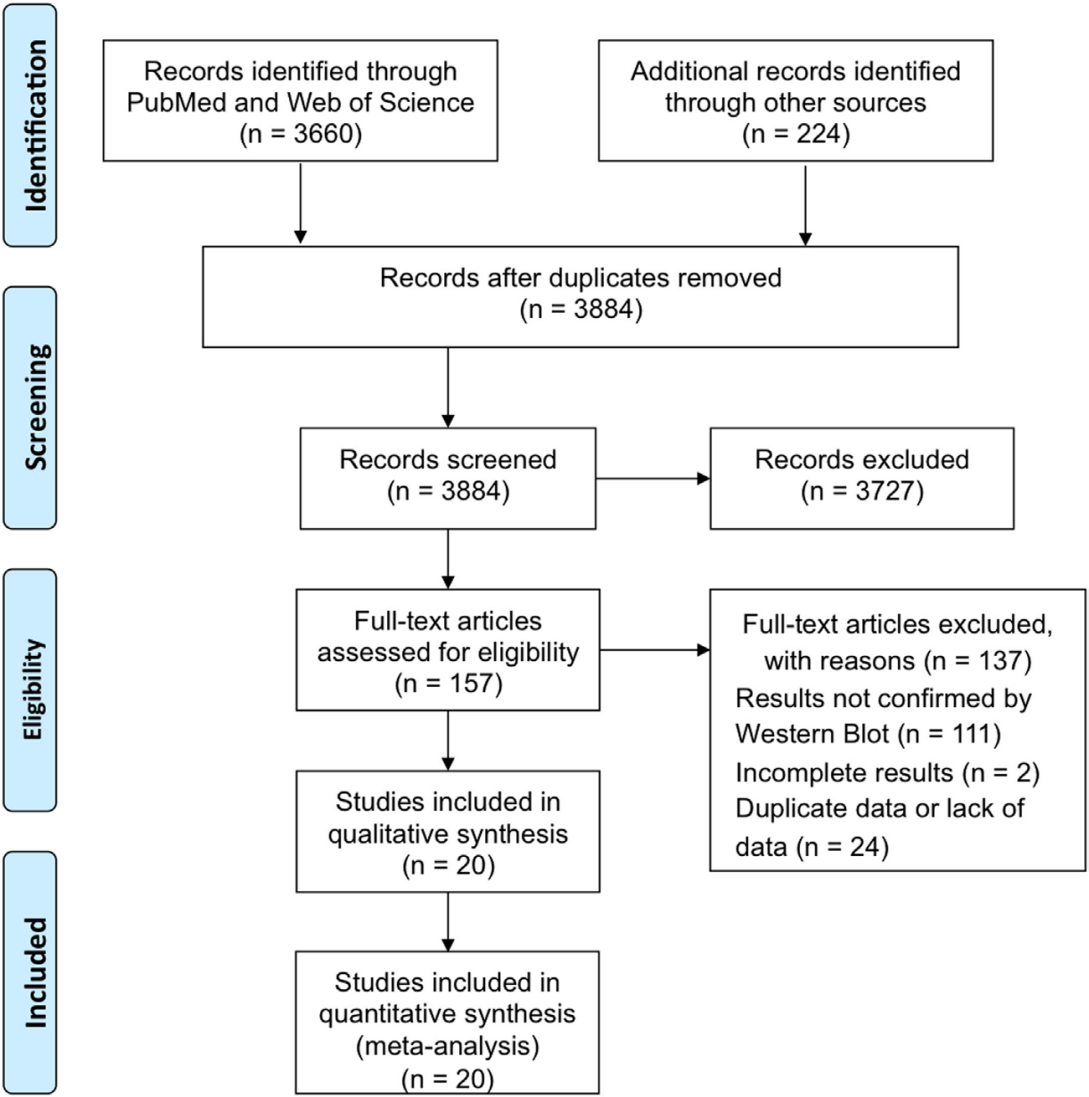
Flow chart of the screening process of the study.

The procedure of study selection and numbers of included and excluded studies is shown in Figure 1.

### Data Extraction

Two independent researchers (Bin Su and Zhiying Liu) extract data of each study into a spreadsheet. The following information was extracted from each selected study: information about the article (name of the first author, journal title, and the publication year), information about the RT (assay type, specimen type, and number of the test samples), information about the HIV-infected patients, and the performance outcomes, such as sensitivity, specificity, PPV, and NPV or other relevant data, to construct 2 × 2 tables of test results.

If there were several RTs performed on the blood samples from the same subjective populations in a study, just one RT could be included in a certain meta-analysis to ensure the independence of the included studies in one meta-analysis.

The quality of individual studies was rated by two independent reviewers (Xinchao Liu and Yaokai Chen) using a Quality Assessment of Diagnostic Accuracy Studies (QUADAS) scale (17).

### Statistical Analysis

Diagnostic meta-analysis was conducted by using command “midas” in StataSE 12.0, where the command required the number of observations of greater than or equal to 4. The presence of between-study heterogeneity when combining the study results was assessed using the chi-square-based *Q*-test of heterogeneity and the *I*^2^ statistic (18). Heterogeneity was considered as moderate to large when *P* < 0.1 for *Q*-test or *I*^2^ > 50%. Due to the paucity of trials in each comparison, we relied primarily on the *I*^2^ statistic. Fixed-effect models were adopted to combine the performance measures derived from studies not presenting moderate to large heterogeneity, assuming that all differences in observed effects were due to sampling error, and that there was one true effect size (i.e., the fixed effect) which underlined all the studies in the analysis. Otherwise, meta-analyses were conducted by random-effects models, because the true effect could vary from study to study and the true effect sizes were assumed to represent a random sample (i.e., the random effects) of effect sizes of individual studies.

The combined performance indexes included the pooled sensitivity, specificity, summary receiver operating characteristic (SROC) curve, and diagnostic odds ratio (DOR) (19). In a single diagnostic test, sensitivity and specificity are defined as TP/(TP + FN) and TN/(TN + FP), respectively, where TP, FP, FN, and TN represent the number of true positives, false positives, false negatives, and true negatives, respectively. Then, DOR is defined as:

$$\text{DOR}=\frac{\text{TP}}{\text{FN}}/\frac{\text{FP}}{\text{TN}}=\frac{\text{sensitivity}}{1-\text{sensitivity}}/\frac{1-\text{specificity}}{\text{specificity}}.$$

DOR is more suitable to compare diagnostic performance for two or more diagnostic tests. A DOR value ranges from 0 to infinity, with a higher value indicating a better discriminatory performance. DOR value of 1 means that a test does not discriminate between patients with and without the disorder. DOR also equals to the ratio of positive likelihood (LR+) and negative likelihood ratio (LR−), where a diagnostic test with LR+ >10 and LR− <0.1 (i.e. DOR > 100) is considered to be excellent discriminatory performance.

For those more used RT assays, one meta-analysis would be conducted on each of them to evaluate their performances, while one meta-analysis would be conducted on a mix of different assays that were just used in one or two RTs.

## Results

### General Study Information

The search strategy identified 3,884 papers from electronic databases and other sources in total. By reviewing the titles and abstracts of these papers, 3,727 irrelevant papers were ruled out. Of the remaining 157 studies that assessed rapid HIV testing, 111 articles without final retest results confirmed by WB were excluded after reviewing abstracts; 2 studies were excluded after detailed reviewing of the full text, because they did not provide sufficient information to construct the diagnostic 2 × 2 tables. Another 24 articles were excluded due to duplicate data or lack of data. Therefore, 20 papers involving 27,343 fresh specimens for RTs were included in the meta-analyses (Figure 1).

Among the 27,343 specimens from 20 studies, 6,753 specimens were HIV-positive and 20,590 were HIV-negative by WB. The rapid HIV antibody detection assays used in these studies included Capillus HIV-1/HIV-2^®^ (Trinity Biotech, Ireland or Cambridge Diagnostics, Galway, Ireland), Determine HIV-1/2^®^ (Abbott Laboratories, Tokyo, Japan or Inverness Medical Japan Co. Ltd., Japan), Uni-Gold HIV test^®^ (Trinity Biotech, Inc., Wicklow, Ireland), and other independent assays. Combinations of two independent assays were also used in six studies. A summary of the studies and their key features is listed in Table 1.

**Table 1:** Characteristics and outcomes of studies included in the meta-analysis.

First author	Year	Country/region	QUADAS score	Number of sample	Subject	Assays	Manufacturer	Sensitivity	Specificity	PPV	NPV
Urassa (20)	2002	Tanzania	10	1,412	Blood donors/antenatal clinic patients	Capillus HIV-1/HIV-2^®^	Trinity Biotech, Ireland	1.0000	0.9870	0.9672	1.0000
						Determine HIV-1/2^®^	Abbott Laboratories, Tokyo, Japan	1.0000	0.9790	0.9457	1.0000
						Serocard HIV	Trinity Biotech, Ireland	1.0000	0.9820	0.9551	1.0000
						Serocard HIV and Determine HIV-1/2^®^	Trinity Biotech, Ireland	1.0000	0.9930	0.9820	1.0000
							Abbott Laboratories, Tokyo, Japan				

Phillips (21)	2000	Asia, Africa, and the America	8	241	NA	Capillus HIV-1/2	Cambridge Diagnostics, Galway, Ireland	0.9885	1.0000	1.0000	0.9718
						HIV Chek 1 + 2	Ortho Diagnostic Systems, Raritan, NJ, USA	1.0000	1.0000	1.0000	1.0000

Lien (22)	2000	Vietnam	13	347	Patients	Capillus^®^ HIV-1/HIV-2	Cambridge Diagnostics, Galway, Ireland	1.0000	0.9960	0.9899	1.0000
						Determine™ HIV-1/2	Abbott Laboratories, Inc., Abbott Park, IL, USA	1.0000	0.9960	0.9899	1.0000
						Serodia^®^ HIV	Fujirebio, Tokyo, Japan	1.0000	1.0000	1.0000	1.0000

Kacem (23)	2001	Ivory Coast	12	1,216	Pregnant women	Capillus HIV-1/HIV-2^®^	Cambridge Diagnostics, Galway, Ireland	1.0000	0.9970	0.9953	1.0000
						Determine HIV-1/2^®^	Abbott Laboratories, Tokyo, Japan	1.0000	0.9940	0.9906	1.0000
						Genie II HIV-1/HIV-2	Sanofi Diagnostics Pasteur, Marne la Coquette, France	1.0000	1.0000	1.0000	1.0000

Waheed (24)	2013	Pakistan	10	472	NA	Capillus HIV-1/HIV-2^®^	Rapid Test Kit Trinity Biotech, USA	0.9460	1.0000	1.0000	0.9270
						SD Bioline	Stander Diagnostic Inc., Korea	1.0000	0.9840	0.9890	1.0000

Ferreira Junior (25)	2005	Brazil	9	1,100	NA	Capillus HIV-1/HIV-2^®^	Trinity Biotech, Ireland	1.0000	1.0000	1.0000	1.0000
						Determine HIV-1/2^®^	Abbott Laboratories, USA	1.0000	0.9989	0.9955	1.0000
						Uni-Gold HIV test^®^	Trinity Biotech, Ireland	1.0000	1.0000	1.0000	1.0000
						Hema-Strip HIV-1/2	Saliva Diagnostic Systems, USA	0.9774	1.0000	1.0000	0.9943

Foglia (26)	2004	Kenyan	9	486	Volunteers	Determine HIV-1/2^®^	Abbott Laboratories, Inc., Abbott Park, IL, USA	1.0000	0.9930	0.9390	1.0000
						Uni-Gold HIV test^®^	Trinity Biotech, Inc., Wicklow, Ireland	1.0000	1.0000	1.0000	1.0000

Rouet (27)	2004	West Africa	13	605	ANRS 1201/1202 Ditrame Plus cohort	Determine HIV-1/2	Abbott Laboratories, Abbott Park, IL, USA	1.0000	0.9840	0.8450	1.0000
						Genie II HIV-1/HIV-2	Bio-Rad, Marnes-La-Coquette, France	1.0000	1.0000	0.8450	1.0000
Holguín (28)	2004	Spain	10	111	HIV-infected patients	Determine HIV-1/2^®^	Inverness Medical Japan Co. Ltd., Japan	1.0000	1.0000	1.0000	1.0000
						Multispot HIV-1/2	Bio-Rad Laboratories	1.0000	1.0000	1.0000	1.0000

Delaney (29)	2011	United States	13	3,775	High risk MSMs/high risk for HIV infection	Uni-Gold HIV test^®^	Trinity Biotech, Inc., Wicklow, Ireland	0.9719	0.9994	0.9889	0.9986
				4,052		Clearview COMPLETE	NA	0.9799	0.9995	0.9900	0.9990

Iqbal (30)	2012	India	11	180	HIV-infected patients	Enzaids Duet HIV	NA	1.0000	1.0000	1.0000	1.0000

Motta (31)	2013	Brazil	13	972	HIV-infected patients/pregnant women/volunteers	Labtest HIV	Labtest Diagnóstica, Lagoa Santa, Brazil	0.9930	1.0000	1.0000	0.9990

Cappello (32)	2013	Nigeria and United States	13	645	Volunteers	Fingerstick whole blood	Alere Inc., Orlando, FL, USA	0.9980	1.0000	1.0000	0.9990

Santos (33)	2011	Angola	11	435	NA	VIKIA HIV1/2	bioMerieux, Marcy-l’Etoile, France	1.0000	1.0000	1.0000	1.0000

Wesolowski (34)	2011	United States	13	3,598	HIV-infected patients	Multispot HIV-1 and Multispot HIV-2	NA	0.9995	0.9940	0.9957	0.9993

Galiwango (35)	2013	Uganda	11	2,520	Volunteers	HIV-1/2 Stat-Pak^®^ Dipstick and Uni-Gold™ HIV	Chembio Diagnostic Systems, Medford, NY, USA	0.9970	0.9970	0.9940	0.9980
							Trinity Biotech, Bray, Ireland				

Lyamuya (36)	2009	Tanzania	12	1,433	Patients/pregnant women/voluntary counseling/blood donors	Determine™ HIV-1/2 and Uni-Gold™ HIV-1/2	Inverness Medical Japan Co. Ltd., Japan	1.0000	1.0000	1.0000	1.0000
							Trinity Biotech, Wicklow, Ireland				

Menard (37)	2005	Central African Republic	12	286	Volunteers	Determine HIV-1/2^®^ and Uni-Gold HIV test^®^	Abbott Laboratories^®^, Tokyo, Japan	1.0000	0.9850	0.9800	1.0000
							Trinity Biotech^®^, Dublin, Ireland				

Manak (38)	2015	Nigeria	13	3,187	Volunteers	Determine (DT) HIV-1/2	Alere Medical Company, Limited, Chiba, Japan	0.9850	0.9870	0.8930	0.9980
						HIV-1/2 Stat-Pak (SP) dipstick	Chembio Diagnostic Systems, Medford, NY, USA	0.9810	0.9980	0.9810	0.9980
						Determine (DT) HIV-1/2 and Uni-Gold (UG) HIV	Alere Medical Company, Limited, Chiba, Japan	0.9810	0.9997	0.9970	0.9980
							Trinity Biotech^®^, Dublin, Ireland				

Mbachu (13)	2015	Nigeria	12	166	Pregnant women	Determine HIV-1/2^®^ and Uni-Gold HIV	Abbott Laboratories^®^, Tokyo, Japan	0.9500	1.0000	0.9930	1.0000
							Trinity Biotech^®^, Dublin, Ireland				

*PPV, positive predictive value; NPV, negative predictive value; QUADAS, Quality Assessment of Diagnostic Accuracy Studies*.

### Diagnostic Performance for a Single RT Assay

**Figure 2:**
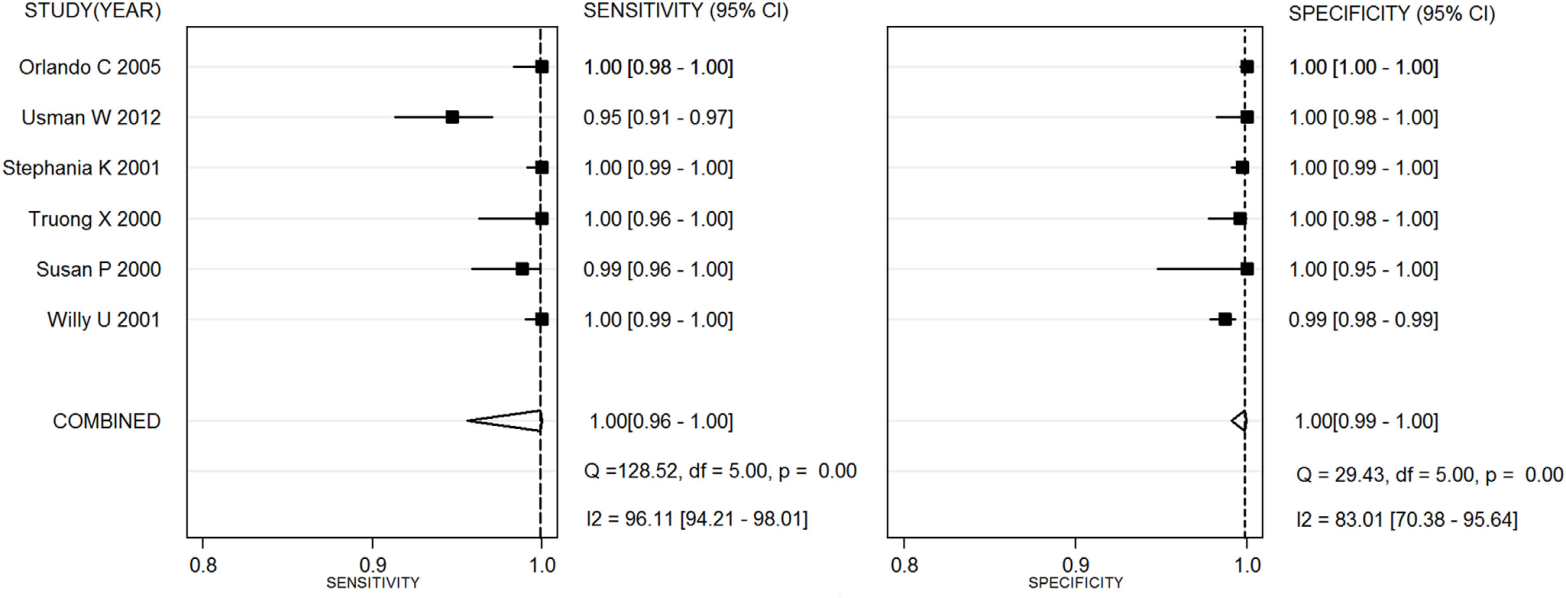
Forest plots for rapid test assay Capillus HIV-1/HIV-2^®^.

**Figure 3:**
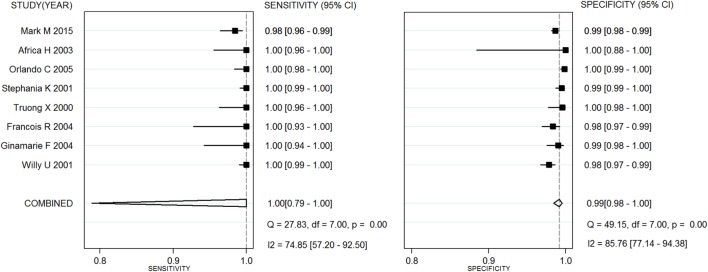
Forest plots for rapid test assay Determine HIV-1/2^®^.

Capillus HIV-1/HIV-2^®^, Determine HIV-1/2^®^, and Uni-Gold HIV test^®^ were used in six studies (20–25), eight studies (26–28), and three studies (25, 26, 29), respectively. Two meta-analyses were conducted on RTs with Capillus HIV-1/HIV-2^®^ and Determine HIV-1/2^®^. The statistic *I*^2^s in the heterogeneity tests were 96% for the pooled sensitivity and 83% for the pooled specificity, 75% and 86% among the included studies with regard to Capillus HIV-1/HIV-2^®^ and Determine HIV-1/2^®^, indicating moderate to large heterogeneity among the included studies. Therefore, random-effects models were chosen for the meta-analyses. In addition, 14 other RT assays, such as Serocard HIV, HIV Chek 1 + 2, Serodia HIV, and Genie II HIV-1/HIV-2, were used in 14 independent studies (20–25). These RTs with mixed assays were included into one single meta-analysis. Performance measures for evaluating these assays are listed in Table 2; Figures 2 and 3 depict their forest plots.

**Table 2:** Diagnostic performance for three commonly used RT assays.

Assay	Pooled sensitivity (95% CI)	Pooled specificity (95% CI)	Area under SROC curve (95% CI)	DOR (95% CI)
Capillus HIV-1/HIV-2^®^	0.999 (0.956–1.000)	0.999 (0.991–1.000)	1.00 (0.99–1.00)	1.0 × 10^6^ (2.6 × 10^4^, 3.9 × 10^7^)
Determine HIV-1/2^®^	1.000 (0.789–1.000)	0.992 (0.985–0.996)	1.00 (0.99–1.00)	1.8 × 10^6^ (406.049, 7.8 × 10^9^)
Mixed assays	0.998 (0.991–1.000)	0.999 (0.995–1.000)	1.00 (0.99–1.00)	7.2 × 10^5^ (8.1 × 10^4^- 6.3 × 10^6^)

*SROC, summary receiver operating characteristic; DOR, diagnostic odds ratio; CI, confidence interval; RT, rapid test*.

### Diagnostic Performance for RT Assay Combinations

There were seven kinds of RT assay serial combinations (34–37), involving Determine HIV-1/2^®^, Uni-Gold HIV test^®^, HIV-1/2 Stat-Pak^®^ Dipstick, Multispot HIV-1 and HIV-2, and Serocard HIV. The commonly used serial combinations are Determine HIV-1/2 and Uni-Gold HIV. The pooled sensitivity, specificity, and area under the SROC curve were 0.998 (95% CI, 0.991–1.000), 0.998 (95% CI, 0.994–0.999), and 1.00 (95% CI 0.99–1.00), respectively. DOR was 2.7 × 10^5^ with 95% CI of 9.0 × 10^4^–8.1 × 10^5^.

## Discussion

The prevention and control of HIV/AIDS as a chronic infectious disease should focus on timely detection and treatment. To further increase the coverage and availability of testing, traditional testing methods will not suffice (11, 39). To achieve widespread testing coverage and rapid result acquisition, RT is a promising diagnostic approach that should be promoted. Though in many studies specificity and sensitivity were reportedly high, single studies were unable to give sufficient statistical power (20, 37). While global guidance from WHO promotes a RT strategy, it has not been successfully implemented globally. One hypothesized reason for the lag in implementation is the inadequate research evaluating the effectiveness of RTs as an alternative to WB as a confirmatory HIV test. Moreover, it is difficult to validate trustworthy testing algorithms using different RTs with varying sensitivity and specificity. Currently, RT reagents can be used for blood (including whole blood, serum, or plasma) and saliva. Several surveys conclude that RT reagents have lower specificity and higher false positive rate in saliva than in blood (40). Therefore, we conducted a meta-analysis to evaluate the specificity and sensitivity of blood-based RT compared with the WB assay in terms of pooled sensitivity, specificity by meta-analysis in a large number of samples. All the studies with appropriate reference (confirmed with WB) reported excellent estimates of sensitivity and specificity. We evaluated the performance of these RT strategies in the context of multiple diagnostic algorithms. Our meta-analysis evaluated the effectiveness of using different FDA-approved RTs on diagnostic accuracy. Areas under the SROC curves for the two most popular rapid assays (Capillus HIV-1/HIV-2 and Determine HIV-1/2) were all above 0.99 with sensitivities above 99.9%. In addition to the most popular RTs, 15 other kits have been evaluated in 15 studies (see mixed assays in Table 2). The pooled sensitivity, specificity, and area under the SROC curve of these assays were 0.998, 0.999, and 1.00, respectively, which demonstrates blood-based rapid HIV test has comparable accuracy to WB for HIV early therapy.

HIV screening *via* successive or simultaneous RT reagents has been widely adopted in Africa (41). Two successive RT reagents have lower costs than simultaneous RT reagents and are widely used for HIV screening (24). A study in Tanzania indicates that a good pair in combination is Korean SD and US Abbott Determine (20). SD can be used for screening and Determine can be used to recheck positive results. Both the sensitivity and specificity of this combination can reach up to 100% (23). Our meta-analysis also studied serial testing strategies (the second test is done only if the first test is positive). Overall, the pooled sensitivity and specificity were 0.998 and 0.998, respectively. Therefore, a serial two-step testing strategy has comparable accuracy to single test strategies.

The FDA regulation for manufacturers seeking licensure of tests recommends that the lower bound of the one-sided 95% confidence interval for sensitivity and specificity exceed 98% (12). Our review suggests that blood-based RT have high diagnostic accuracy, with comparable estimates when using a two-step or single testing strategy. It leads to early diagnosis and treatment of HIV and better clinical results. These data have the potential to change recommendations on voluntary counseling and testing from using originally ELISA based testing to RTs, and furthermore to replace the confirmatory WB test for HIV early therapy at the same day of detection. Particularly in countries and regions with high HIV/AIDS prevalence, timely actions should be taken to develop the relevant policies, technical protocols, and quality assurance systems to ensure the widespread implementation of RT.

Although the sensitivity and specificity of RT reagents both exceed 99.5%, they could be compromised due to unstandardized operations in non-laboratory settings (42, 43). The sensitivity of RT can be reduced in the absence of quality assurance and evaluation system (21). Unstandardized operations may lead RT false negative rate of up to 5.4% (29). RT test inevitably faces other challenges, such as inability of rechecking the same sample, and relatively low sensitivity for early HIV infection (42).

Our meta-analysis has several strengths. Algorithms either using serial RT testing strategies or single FDA-approved RT have been proved with satisfactory results. In addition, there has been an expansion in suitable specimen types (finger stick whole blood). We performed a comprehensive search of sources to identify studies that adopt different kinds of RTs. Several meta-analyses addressing the efficacy of so-called “rapid HIV testing” have been published recently, some of which focused on the fourth-generation ELISA test (Ag/Ab combination) that takes several hours to get testing result instead of “real” rapid HIV tests (43–45). And small sample sized meta-analysis has showed that rapid HIV voluntary counseling and testing improves the receipt rate of HIV test results among clients who seek HIV counseling and testing (45). Therefore, our current meta-analysis contributed uniquely to the field with greater sample size and trustworthy results. In some countries and regions, traditional tests still prevail, particularly in China. Thus, it is feasible to have RT performed by trained non-medical professionals outside laboratories, which can promote HIV testing services among high risk groups such as MSM population more easily and greatly enhance both the awareness rate and result notification rate of the infected and the coverage of ART.

There also have been several limitations. For example, statistical comparison between subgroups (i.e., different populations) was not possible due to lack of data. Only English language studies were included in this meta-analysis, which may lead to a potential reporting bias. Different areas can determine the combinations based on the performance of reagents, costs, HIV prevalence, and risk behaviors of populations. Especially in low epidemic areas, indicating the need for counselors and clients to understand the limitations of RT positive results and the necessity to receive confirmatory tests. WB confirmatory test can be applied mainly for confirmation of indeterminate result.

Overall, our study indicated that RT would function as well as the WB and RT can and should be accessible and extensively used for HIV early therapy at the same day of detection if it is carried out by standardized management protocols. Quality assurance and quality control of RTs are extremely important and non-trivial in a devolved environment where such practices are not engrained in the culture, thus it is of great importance to acquire the knowledge and skills required for conducting quality control at a RT site. The time is ripe for RTs to play a more central role in increasing timely identification of HIV infections.

## Conclusion

Our meta-analysis results may provide evidenced-based support for substituting rapid test for WB. Blood-based rapid HIV tests have comparable sensitivity and specificity to WB for HIV early therapy.

## Author Contributions

Study conception and design: HW, HC, and XH. Acquisition of data: XL, JC, BS, ZL, XH, and YC. Preparation of figures: HC, ZL, BS, XL, and WX. Preparation of tables: HC, HX, and AS. Writing and revision of the manuscript: XL, JC, YC, and XH. All the authors have given approval to the final version of the manuscript.

## Conflict of Interest Statement

The authors have declared that no competing interests exist and no manufacturers have funded this study.
